# P-2006. Development of a Machine Learning Model for the Accurate Diagnosis of Common Tropical Febrile Illnesses

**DOI:** 10.1093/ofid/ofaf695.2170

**Published:** 2026-01-11

**Authors:** C Shravya, R Rajalakshmi, Muhammed Rashid, Girish Thunga, Vijayanarayana Kunhikatta, Muralidhar Varma, Vasudha Devi, Raviraj V Acharya, K N Shivshankar, Ashwini Amin, Dinesh Acharya U, Sohil khan

**Affiliations:** Manipal College of Pharmaceutical Sciences, Manipal Academy of Higher Education, Manipal, Manipal, Karnataka, India; Manipal College of Pharmaceutical Sciences, Manipal, Karnataka, India; Federation of Clinical Pharmacists in India, Bangalore, Karnataka, India; Manipal College of Pharmaceutical Sciences, Manipal, Karnataka, India; Manipal College of Pharmaceutical Sciences, Manipal Academy of Higher Education, Manipal, Manipal, Karnataka, India; Kasturba Medical College, MAHE, Manipal, Manipal, Karnataka, India; Kasturba Medical College, MAHE, Manipal, Manipal, Karnataka, India; Kasturba Medical College, MAHE, Manipal, Manipal, Karnataka, India; Kasturba Medical College, MAHE, Manipal, Manipal, Karnataka, India; Manipal Institute of Technology, Manipal, Karnataka, India; Manipal Institute of Technology, Manipal, Karnataka, India; Manipal College of Pharmaceutical Sciences, Manipal Academy of Higher Education, Manipal, Manipal, Karnataka, India

## Abstract

**Background:**

Acute febrile illnesses (AFIs) such as dengue, malaria, scrub typhus, leptospirosis etc, prevalent in tropical regions, account for 17% of the global disease burden. Overlapping clinical features and limitations of current diagnostic methods—including false positives, sensitivity/specificity issues made the diagnosis complicated. With increasing digital integration in healthcare, artificial intelligence (AI) offers a promising solution for improving diagnostic accuracy and efficiency. Our study aimed to develop an AI-based tool to aid differential diagnosis of AFIs and support clinical decision-making.

**Fig 1:**
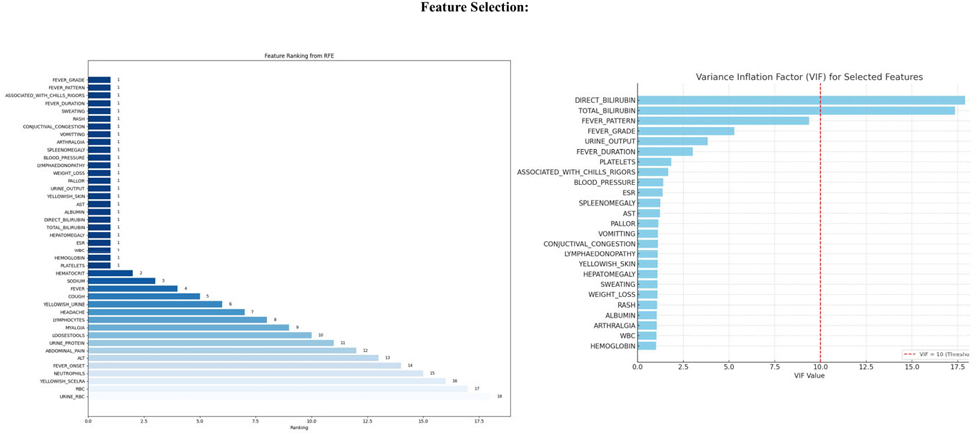
Features selected through Recursive Elimination and Multicollinearity Based on Recursive_Feature_Elimination (RFE), a set of high-ranking features was initially identified. These features were then subjected to multicollinearity assessment to eliminate redundant variables with strong linear relationships. The final set of 20 non-collinear, clinically relevant features was selected for model development and is presented in Fig_1

**Fig 2:**
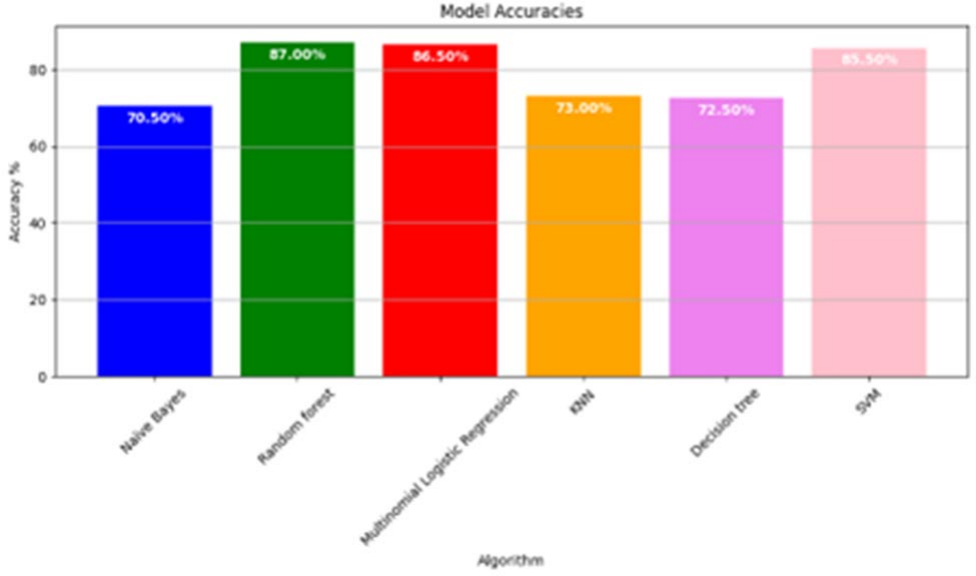
Accuracy of the developed models

**Methods:**

A retrospective cross-sectional study analyzed records of 800 patients (200 per disease). Clinical data were extracted and preprocessed (cleaning, scaling, imputation). Feature selection was conducted using Recursive_Feature_Elimination (RFE) and multicollinearity assessment. Models were developed using supervised machine learning algorithms— Random_Forest(RF), Naïve_Bayes(NB), Logistic_Regression(LR), Support_Vector_Machine(SVM), K-Nearest_Neighbors(KNN), and Decision_Tree(DT). A stacking classifier served as a meta-model. Performance was evaluated using accuracy, precision, recall, and F1-score.

**Fig 3:**
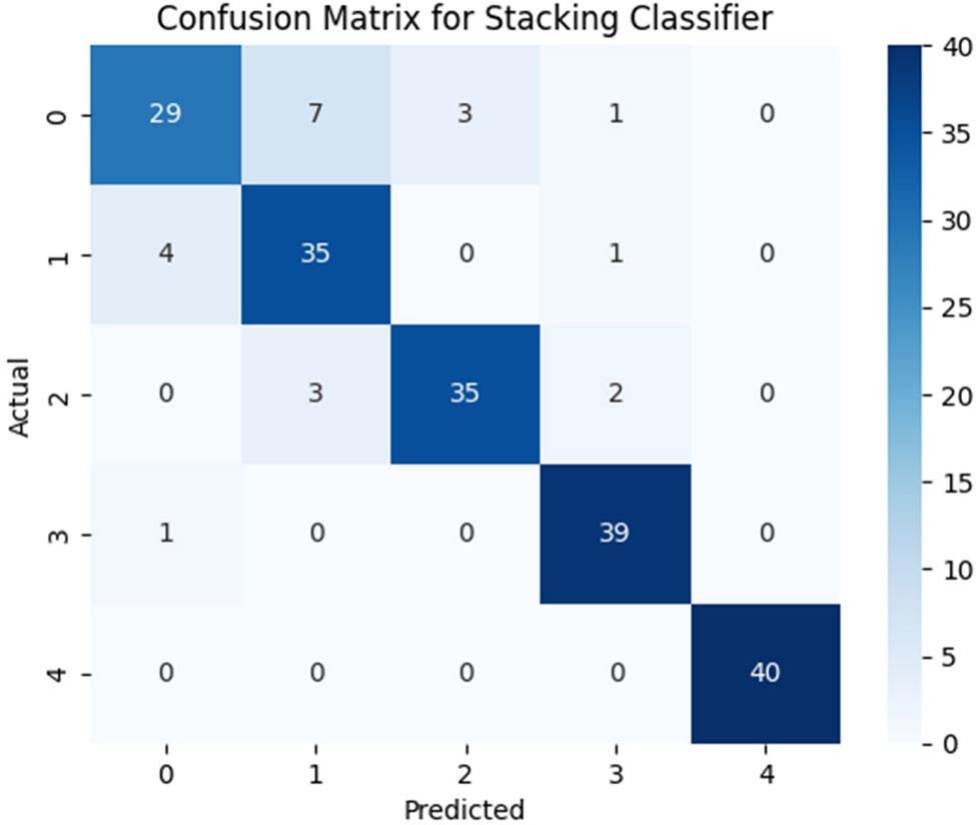
Confusion matrix for Stacking Classifier

**Table 1:**
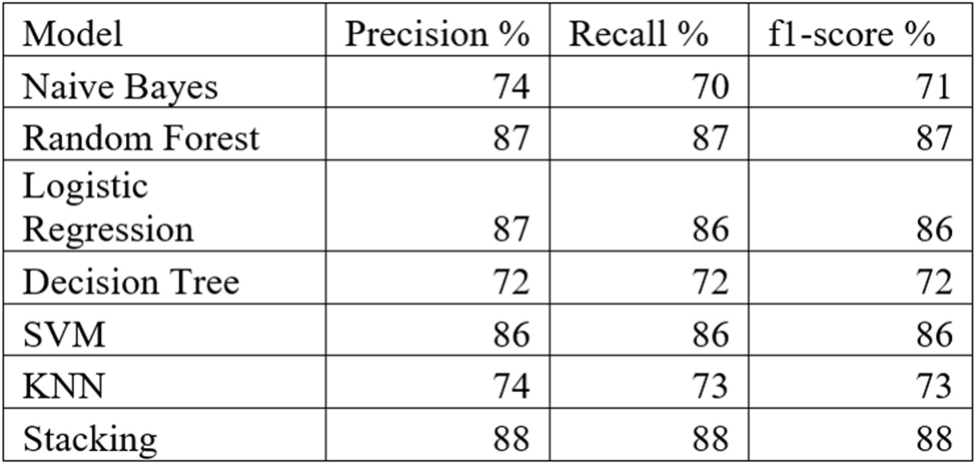
Other performance Metrics

**Results:**

Based on RFE, set of high-ranking features were identified and were then subjected to multicollinearity assessment. The final set of 20 non-collinear, clinically relevant features was selected for model development (presented in Fig_1). Among the developed predictive models, the RF demonstrated highest classification accuracy (87%), followed by LR (86.5%), SVM (85.5%), KNN (73%), DT (72.5%), and NB (70.5%), (shown in Fig_2). Additional performance metrics, including precision, recall, and F1-score, are summarized in Table 1. A stacking classifier, integrating the predictions of all base models, achieved an overall accuracy of 89% on training dataset. The confusion matrix for the stacking model is presented in Fig_3

**Conclusion:**

By integrating clinical data with advanced feature selection techniques, AI-based tool can be the future diagnostic aiding tool for screening the tropical diseases.

**Disclosures:**

All Authors: No reported disclosures

